# Revision of *Ilyphagus* Chamberlin, 1919 (Polychaeta, Flabelligeridae)

**DOI:** 10.3897/zookeys.190.3059

**Published:** 2012-05-04

**Authors:** Sergio I. Salazar-Vallejo

**Affiliations:** 1 El Colegio de la Frontera Sur, CONACYT, Departamento de Ecología Acuática, Chetumal, Quintana Roo, México

**Keywords:** Annelida, *Bradabyssa*, *Diplocirrus*, *Therochaeta*, caruncle, branchial filaments, neurochaetae, taxonomy

## Abstract

*Ilyphagus* Chamberlin, 1919 includes abyssal, fragile benthic species. Most species have large cephalic cages but chaetae are brittle and easily lost which may explain why the original definition included species with a cephalic cage or without it. The type species, *Ilyphagus bythincola* Chamberlin, 1919, together with another species (*Ilyphagus pluto* Chamberlin, 1919) were described as lacking a cephalic cage whereas a third species (*Ilyphagus ascendens* Chamberlin, 1919) was described with one. To clarify this situation, all available type and non-type materials were studied. *Ilyphagus* is redefined to include species with digitiform bodies, abundant filiform papillae and a thin body wall; their neurochaetae are thick, anchylosed aristate spines, and all species have a cephalic cage (in the type species the presence of a cage is inferred from the remaining chaetal scars). *Ilyphagus pluto*, which also lacks a a cephalic cage is determined here to be a holothurian. The redefined genus contains *Ilyphagus bythincola* (incl. *Ilyphagus ascendens*), *Ilyphagus coronatus* Monro, 1939, *Ilyphagus hirsutus* Monro, 1937, and *Ilyphagus wyvillei* (McIntosh, 1885).

## Introduction

Deep-sea animals are often bizarre. By the end of the XIX century, or during the early XX century, the dredged animals collected during deep-sea expeditions were surprising marine zoologists. Trying to cope with the unexpected body patterns, some general features might have been regarded as diagnostic for grouping species that could be more easily studied. This might explain why Chamberlin (1919) proposed a new name for some polychaetes that were regarded as feeding on mud. By combining the Greek words for mud (*Ilys*) and glutton (*phagos*), he established *Ilyphagus*. For its definition, he relied upon three different species: one with a well-developed cephalic cage, and two others apparently deprived of it. Thus, the generic diagnosis was wide enough to include species in either condition, and that concept prevailed in the current definition (Fauchald 1977). The cephalic cage is a distinctive feature for most flabelligerids. It is made up of long chaetae, usually pointing forward and stemming from at least one of the first few chaetigers. Although it has been a useful diagnostic feature to separate flabelligerid genera, it has been enigmatic how a single genus might contain two distinct morphological patterns; one with a cephalic cage and the other without it. Further, the body shape for *Ilyphagus* has been regarded as depressed or even disc-shaped (Fauchald 1977:117). This, in turn, is explained by the collapse of the body due to compression because the body is subcylindrical in life. Dredging or sieving sediments might distort the body shape, because the thin body wall is easily broken, such that the body becomes depressed, or flat.

Monro (1937:305) made some interesting comments on the genus. He essentially indicated two issues: first, that *Ilyphagus* is an abyssal genus, and second, that there was a single species that included the three described by Chamberlin (*Ilyphagus bythincola*, *Ilyphagus pluto*, and *Ilyphagus ascendens*), and his *Ilyphagus hirsutus*. Monro was correct on the first statement, because all species do come from deep water habitats; for the second assertion, however, he failed to acknowledge the differences in papillae development and chaetae. Later, he indicated that all species in the genus were described as lacking a cephalic cage (Monro 1939:131), which was not the case, and that the cephalic cage chaetae may be present, but may be broken during collection. The latter is correct and will be discussed later.

*Ilyphagus* species are poorly known because of their life in deep-water and because they have not been found in hydrothermal vents, cold seeps or whale remains, which have recently received a lot of attention. Further, the species of *Ilyphagus* have very low abundances, most species being known from a single or few specimens. They might live in the sediment-water interface, with the body barely covered by sediment and the very long cephalic cage chaetae are probably used as an anti-predation device.

Because there are problems in the definition for the genus and for the species, the purpose of this revision was to study all material available and currently regarded as belonging in *Ilyphagus*. It was expected that this study would result in a redefinition of the genus, a clarification of the diagnostic features, and probably a modification of the included species. The presence of a cephalic cage is confirmed for the genus and after redefining it, it contains four species.

## Methods

All specimens were studied under the stereomicroscope. They were often stained by a few seconds immersion in an oversaturated solution of methyl-green in 70% ethanol which is temporary. Individual chaetae or parapodial rami were observed in compound microscopes. The plates were made by selecting one or by editing several digital pictures of the same objects. The anterior end was dissected to study the head structure and the associated appendages. The materials belong to the following collections.

### Museum and collections acronyms

LACM-AHF Museum of Natural History, Los Angeles, Allan Hancock Foundation Polychaete Collection.

NHML The Natural History Museum, London.

SIORAS Shirshov Institute of Oceanology, Russian Academy of Sciences, Moscow.

USNM National Museum of Natural History, Smithsonian Institution, Washington.

ZIRAS Zoological Institute, Russian Academy of Sciences, Sankt-Peterburg.

ZMH Zoologisches Museum und Institut, Universitat Hamburg, Hamburg.

## Results

### Morphology

**Body**.The body is sausage-shaped; it may be short and oval, or cigar-shaped, and may even be swollen anteriorly. In some species, juveniles have thinner bodies and may be confused with some species of *Bradabyssa* Hartman, 1967; however, in *Ilyphagus*, the body wall is thin and covered by delicate, long papillae, which are not embedded by the tunic, resulting in a pilose surface. The papillae are filiform, barely swollen distally, if any at all, and are often covered by fine sediment particles. Once the excessive sediment is removed, individual papillae might have a thin or a thick layer of adherent sediment particles. Parapodia are poorly developed and the brittle chaetae emerge from the body wall. Gonopodial lobes are difficult to detect due to the abundant papillae; when they are well developed, they can be visible in chaetigers 5–6.

**Cephalic cage**. The species belonging in *Ilyphagus* carry very long chaetae in the first 1–2 chaetigers. The first parapodia are markedly displaced dorsally, with both parapodial rami very close to each other and approaching the middorsal line. The second chaetiger has notopodia more dorsally displaced than the first but they are quite separated from the corresponding neuropodia. The second chaetiger’s notochaetae are arranged in an oblique line or in a bundle, whereas the first notochaetae are more frontally located. Further, these notochaetae are often the longest, being as long as the whole body, or even longer; this chaetal length is remarkable among the family and among all Polychaeta. Sometimes, these long notochaetae can appear spirally twisted, as in the maldanid polychaete *Nicomache maculata* Arwidsson (Kennedy and Kryvi 1978). However, the chaetae in *Ilyphagus*, instead of being formed by spirally twisted fibers, have parallel microvilli and the external surface has a series of constrictions along the shaft which slowly rotate towards the tip. Nevertheless, these spiral chaetae are brittle and seldom available for observation, such that it is unknown how widespread they are, or if the finding detailed below is based upon accidental growth. All cephalic cage chaetae can be broken off, but their presence can be determined by chaetal scars or holes through the body wall, or by making a longitudinal dissection along the anterior end, if there are several specimens available and papillae cover chaetal scars. A large fan of companion chaetae is visible where the notochaetae of the first chaetiger are present.

**Body chaetae**. Notochaetae are multiarticulated, at least distally; there are usually 1–3 notochaetae per bundle and often one has long articles, whereas the others may have short articles only basally or throughout the chaeta. Neurochaetae are thicker than notochaetae; those present in the first chaetiger, or first few chaetigers, may be multiarticulated as well. In the former case, there are some chaetigers with transitional chaetae. They have long articles throughout the chaetae in the second chaetiger, but in following chaetigers the articulated region is progressively reduced, such that it becomes restricted to a short distal region. From chaetigers 2–4, anchylosed aristate spines are present. Neurochaetae have basal transverse marks, but these marks become slightly oblique beyond the median region. The distal region of the neurochaetae is delicate, often hyaline, and aristate; in one species, it is hirsute which is due to the rupture and exposure of the abundant oblique fibers .

**Anterior end**. The anterior end carries two large thick palps and several thick branchial filaments, arranged in 1–2 concentric rows which often resemble a horse-shoe pattern. The nephridial lobes are difficult to find, because they are located on the inner side of the branchial row at about the centre of the branchial plate. The species are apparently devoid of eyes and caruncle; the lack of a caruncle is unexpected because it has been found in all other flabelligerid genera. Although the underlying prostomial wall may be depressed, the ciliary bands may be present; however, because the preservation fluids penetrate slowly, these bands may be difficult to detect. Histological or SEM observations would be required to determine whether the expected bands of cilia are absent, or if they have been reduced.

## Systematics

### Class Polychaeta Grube, 1850. Order Flabelligerida Pettibone, 1982. Family Flabelligeridae de Saint-Joseph, 1894

#### Ilyphagus

Chamberlin, 1919 restricted

http://species-id.net/wiki/Ilyphagus

Ilyphagus Chamberlin, 1919:402; Hartman 1965:177; Fauchald 1977:117.

##### Type species.

*Ilyphagus bythincola* Chamberlin, 1919, by original designation.

##### Diagnosis (emended):

Body digitiform, rounded at both ends, densely covered by thin, abundant papillae. Body wall thin. Cephalic cage well developed; notochaetae dorsal, arranged in transverse rows. Parapodia biramous, inconspicuous. Notochaetae multiarticulated capillaries; neurochaetae thicker, anchylosed aristate spines.

##### Remarks.

The type species, *Ilyphagus bythincola* Chamberlin, 1919, originally described as lacking cephalic cage, in reality has one but the chaetae are broken and only their embedded bases or some chaetal scars are left. The other species described as lacking a cephalic cage, *Ilyphagus pluto* Chamberlin, 1919, is not a polychaete but an abyssal holothurian which belongs to the synallactid genus *Meseres* (identified by the late Dr. Cynthia Ahearn, USNM). The inclusion of *Ilyphagus pluto* allowed a rather broad concept for body shape and chaetal patterns, because this species is rather cylindrical and completely lacks a cephalic cage. Further, Chamberlin (1919) emphasized the lack of large, wide papillae that resemble tubercles, as those found in some species of *Brada*. Thus, he recognized the difference and restricted the inclusion to those species lacking thick papillae (or tubercles).

*Ilyphagus* and *Bradabyssa* Hartman, 1967 are closely allied (Salazar-Vallejo et al. 2008) because they have anchylosed neurospines which are basally annulated (anchylosed short articles) and distally hyaline, tapering into an arista (hence aristate spines). However, there are four main differences between these genera. First, the relative position of the cephalic cage chaetae: they are arranged as transverse dorsal rows in *Ilyphagus*, whereas in *Bradabyssa* they are lateral, fewer and smaller. Second, the development of the body wall: most *Bradabyssa* species have a thick muscular body wall, whereas in *Ilyphagus* species it is reduced with poorly-developed muscle layers. Third, the branchial features: in *Ilyphagus* there are a few thick branchial filaments arranged in a horse-shoe pattern, whereas in *Bradabyssa* they are abundant and medially separated by the caruncle into two half-moon shaped groups. Fourth, and derived from the latter: the species of *Bradabyssa* have a well-developed caruncle whereas in *Ilyphagus* it is reduced or absent.

##### Species included.

Besides the type species, *Ilyphagus bythincola* Chamberlin, 1919 from the Eastern Pacific (including *Ilyphagus ascendens* Chamberlin, 1919), the genus contains *Ilyphagus coronatus* Monro, 1939 from the Antarctic Ocean, *Ilyphagus hirsutus* Monro, 1937 from the Central Indian Ocean, and *Ilyphagus wyvillei* (McIntosh, 1885) from the Antarctic Ocean.

There are several species that have been previously placed in *Ilyphagus* but belong elsewhere. Thus, *Ilyphagus antarcticus* Hartman, 1978, *Ilyphagus ilyvestis* Hartman, 1960 and *Ilyphagus minutus* Amoureux, 1986 belong in *Bradabyssa*, *Ilyphagus caudatus* Rioja, 1963 belongs in *Therochaeta* Chamberlin, 1919, and *Ilyphagus octobranchus* Hartman, 1965 belongs in *Diplocirrus* Haase, 1915 as emphasized elsewhere (Day 1973, Salazar-Vallejo et al. 2008, Salazar-Vallejo and Buzhinskaja 2011). Lastly, as indicated above, *Ilyphagus pluto* Chamberlin, 1919 is not a polychaete but an holothurian.

##### Distribution.

The species of this genus have representatives living in deep to very deep sea sediments (1260–7000 m), from the Pacific, Indian and Antarctic Oceans.

##### Key to species of *Ilyphagus* Chamberlin, 1919 restricted

**Table:** 

1	Body short, about three times longer than wide	2
–	Body cigar-shaped, more than five times longer than wide	3
2(1)	Neurochaetae markedly hirsute subdistally (oblique fibers exposed); chaetiger 1 with 3–4 neurochaetae per side	*Ilyphagus hirsutus* Monro, 1937
–	Neurochaetae barely hirsute or smooth subdistally; chaetiger 1 with about 8 neurochaetae per side	*Ilyphagus bythincola* Chamberlin, 1919 partim
3(1)	Body velvety (papillae short); most neurochaetae smooth or barely hirsute	4
–	Body pilose (papillae long); neurochaetae smooth and hirsute (by fracture); up to 14 branchial filaments	*Ilyphagus coronatus* Monro, 1939
4(3)	Chaetiger 1 with about 8 neurochaetae per side; about 40 branchial filaments	*Ilyphagus bythincola* Chamberlin, 1919 partim
–	Chaetiger 1 with 10–12 neurochaetae per side; about 16 branchial filaments	*Ilyphagus wyvillei* (McIntosh, 1885)

#### Ilyphagus bythincola

Chamberlin, 1919

http://species-id.net/wiki/Ilyphagus_bythincola

Figures 1 2

Ilyphagus bythincola Chamberlin, 1919:402–403, pl. 69, Figs 4–9; Hartman 1960:131; Levenstein 1961b:136; Fauchald 1972:224–225.
Ilyphagus ascendens Chamberlin, 1919:403–404; Hartman 1960:131–132.

##### Type material.

**Eastern Pacific Ocean.** Holotype of *Ilyphagus bythincola* (USNM 19748), one paratype (USNM 19384), off Mexico, R/V Albatross, Stat. 3415 (14°46'N, 98°40'W), 1879 fathoms (3438.6 m), 10 Apr. 1891 (paratype two fragments; may belong to the same organism, not measured). Holotype of *Ilyphagus ascendens* (USNM 19735), off Hood Island, Galapagos Islands, 12 miles (19.3 km) SE Ripple Point, R/VAlbatross, Stat. 4649 (01°35'S, 89°30'W), 633 fathoms (1158.4 m), 10 Nov. 1904.

**Figure 1.**
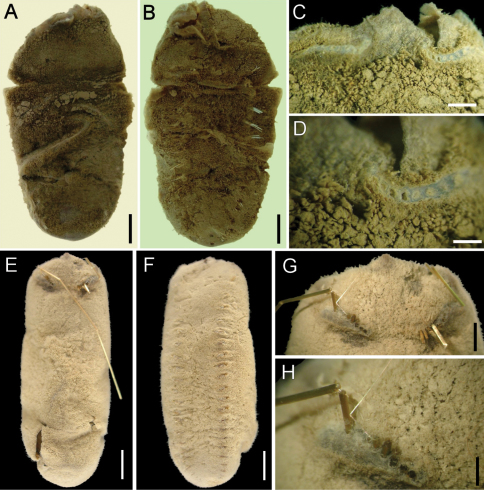
*Ilyphagus bythincola* Chamberlin, 1919. **A** Holotype (USNM 19748) of *Ilyphagus bythincola*, dorsal view **B** Same, ventral view **C** Same, anterior margin, chaetiger 1 **D** Same, close-up showing chaetal cage scars **E** Holotype (USNM 19735) of *Ilyphagus ascendens*, dorsal view **F** Same, ventral view **G** Same, anterior end, dorsal view **H** Same, close-up showing chaetal scars and remaining chaetae. Bars.- **A** 5.9 mm **B** 5.6 mm **C** 1.5 mm **D** 0.8 mm **E–F** 6.8 mm **G** 3 mm **H** 1 mm.

**Figure 2.**
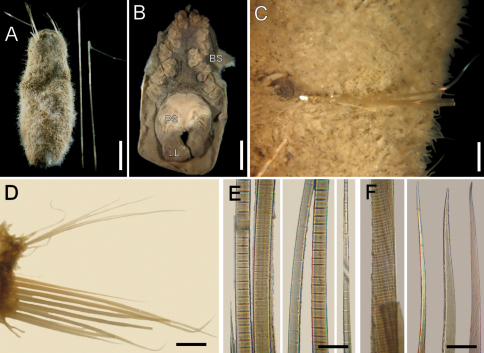
*Ilyphagus bythincola* Chamberlin, 1919, non-type specimens. **A** Complete (USNM-49080), dorsal view **B** Head (SIORAS-unnumb.), frontal view (**BS** branchial scars, **LL** lateral lip, **PS** palp scar) **C** Same, chaetiger 5, ventral view, left neuropodium and gonopodial lobe **D** Same, chaetiger 10, right parapodium **E** Same, basal, medial and distal notochaetal regions **F** Same, median region and tips of neurochaetae. Bars.- **A** 4.9 mm **B** 2.3 mm **C** 0.6 mm **D** 0.7 mm **E** 70 µm **F** 30 µm.

##### Additional material.

**Eastern Pacific Ocean.** One specimen (USNM 49080), off Mexico, R/V Albatross, Stat. 3415 (14°46'N, 98°40'W), 1879 fathoms (3438.6 m), 10 Apr. 1891 complete, K. Fauchald, id. (24 mm long, 9 mm wide, cephalic cage +17 mm long, 22 chaetigers). Two complete specimens (SIORAS-unumb.), R/V Akademik Kurchatov, Stat. 294 (08°23'S, 81°00'W), off Nazca Ridge, 6200–6240 m, Sigsbee trawl, 31 Oct./1 Nov. 1968 (43–75 mm long, 5–8 mm wide, cephalic cage 0–13 mm long (broken), 22–23 chaetigers). Many specimens (SIORAS unnumbered), off Northern Peru, R/V Akademik Kurchatov, Stat. 301 (05°51.7'S, 81°48.8'W), 5300 m, 4 Nov. 1968 (best specimen 48 mm long, 19 mm wide, cephalic cage 21 mm long, 22 chaetigers).

##### Description.

Holotype of *Ilyphagus bythincola* (USNM-19748) damaged, ovoid, body wall broken by compression, depressed (Fig. 1A); 48 mm long, 26 mm wide, cephalic cage chaetae broken, 21 chaetigers. Holotype of *Ilyphagus ascendens* (USNM 19735) with body digitate, ovoid, pointed anteriorly, rounded posteriorly (Fig. 1E); 55 mm long, 20 mm wide, cephalic cage 36 mm long (tips broken), 24 chaetigers. Body surface densely papillated, with fine sediment particles trapped between papillae (Figs 1B, F, 2A); posterior region with longer papillae; each papillae filiform, most with tips pale, some with black tips.

Cephalic cage chaetae broken; scars present in chaetiger 1, short dorsal transverse row with 8–10 chaetal scars per side (Fig. 1C, D); holotype of *Ilyphagus ascendens* with chaetae at least twice as long as body width, perhaps as long as body length (Fig. 1E). Chaetiger 1 (and perhaps 2) involved in the cephalic cage, dorsal; most cephalic cage chaetae broken from the base, 8 notochaetae; neurochaetae lateral, 7 per side, arranged in a short row, transverse (oblique in *Ilyphagus ascendens*, Fig. 1G, H). Non-type specimens with chaetae as long as body length (Fig. 2A). Anterior dorsal margin of first chaetiger papillated. Anterior chaetigers without larger papillae. Anterior end observed in a non-type specimen (SIORAS).

Cephalic hood short, not exposed, margin smooth. Prostomium low, as dark as surrounding region; eyes not seen. Caruncle not seen. Palps large, thick, shorter than branchiae; palp lobes reduced. Lateral lips well developed; ventral and dorsal lips reduced.

Branchiae thick, digitate, in different sizes, sessile on branchial plate, in a horse-shoe pattern (Fig. 2B), one superior single row with four thick larger filaments, and six pairs of lateral filaments arranged in irregular double rows. Largest branchiae longer than palps. Nephridial lobes in branchial plate not seen. Chaetal transition from cephalic cage to body chaetae abrupt (most chaetae broken); first aristate neurospines in chaetiger 3. Gonopodial lobes not seen in holotype; non-type specimens with dark, digitate, small lobes in chaetiger 5 (Fig. 2C), or in chaetigers 5 and 6.

Parapodia slightly developed (Fig. 2D); notopodia without prominent lobe, chaetae emerge from the body wall. Median neuropodia ventrolateral, projected ridges. No additional longer papillae associated with chaetal lobes. Noto- and neuropodia lateroventral, very close to each other.

Median notochaetae arranged in oblique rows, as long as one-fourth or one-fifth of body width, 2–3 per bundle; all notochaetae thin, multiarticulated capillaries, with short articles basally, median-sized medially, longer distally (Fig. 2E). Neurochaetae anchylosed aristate spines, 6–8 per bundle; broken, with short anchylosed articles, arranged in an oblique line. Other chaetal features not examined in holotype. Paratype with noto- and neurochaetae broken; non-type specimens with slightly curved, hyaline, smooth tips (Fig. 2F).

Posterior end rounded; pygidium with anus ventral, without anal cirri.

##### Remarks.

The original body shape was digitate rather than sole-like; this distortion was the result of the sudden change of pressure, especially because of the sediment load in the dredge over its body. The damage resulted in the loss of all cephalic cage chaetae, but chaetal scars are visible in the corresponding position. This damage further compressed the body breaking its wall, and making it appear flat. Other specimens (SIORAS) are long, anteriorly swollen, posteriorly tapered; this is more pronounced among juveniles, and although the number of chaetigers is fixed early in development, counting depends on the presence of chaetae and they are often broken off.

*Ilyphagus bythincola* Chamberlin, 1919 and *Ilyphagus ascendens* Chamberlin, 1919 are herein regarded as synonyms. The latter has cephalic cage chaetae in an oblique row, rather than in a transverse one as in *Ilyphagus bythincola*. This displacement results in a larger area between chaetae and the anterior margin of chaetiger 1. However, because the anterior end of *Ilyphagus bythincola* is severely damaged, and because other body features are similar, there are insufficientdifferences to keep them separate as distinct species.

*Ilyphagus bythincola* resembles *Ilyphagus hirsutus* Monro, 1937, but they differ in the relative number of neurochaetae in chaetiger 1 and in the relative smoothness of neurochaetal tips. Thus, *Ilyphagus bythincola* has about 8 neurochaetae in the first chaetiger, whereas there are 3–4 in *Ilyphagus hirsutus*, and in the former, the neurochaetal tips are mostly smooth or barely hirsute, whereas in *Ilyphagus hirsutus* neurochaetae are markedly hirsute. At the same time, *Ilyphagus bythincola* resembles *Ilyphagus wyvillei* (McIntosh, 1885), but they differ in the relative number of neurochaetae in chaetiger 1 and in the number of branchial filaments. Thus, in *Ilyphagus bythincola* there are 8 neurochaetae per side and about 40 branchial filaments, whereas in *Ilyphagus wyvillei* there are 10–12 neurochaetae and about 16 branchial filaments.

##### Distribution.

Apparently restricted to deep water off southwestern Mexico, to Galapagos and Peru, in 1260–6000 m. There have been two other records for this abyssal species. Levenstein (1961b:137, map), recorded it from the Java Trough, and Kirkegaard (1956:70, Fig. 9) recorded it from the Sunda Trench. The former (ZIRAS-9451) was based on a specimen broken in two, much damaged, collected in 6850 m depth (RV Vitjaz, Stat. 4535, 10º08'S, 107º55'E). It resembles *Ilyphagus bythincola* but better specimens are needed for a complete identification or description. On the other hand, the specimen from the Sunda Trench belongs to an undescribed species in *Bradabyssa*, and will be described elsewhere.

#### Ilyphagus coronatus

Monro, 1939

http://species-id.net/wiki/Ilyphagus_coronatus

Figure 3

Ilyphagus coronatus Monro, 1939:130–131, fig. 19; Hartman 1966:41, pl. 12, Figs 4–6, Hartman 1967:127; Rozbaczylo 1985:159–160.

##### Type material.

**Antarctic Ocean.** Three syntypes of *Ilyphagus coronatus* Monro, 1939 (NHML-1941.3.3.99–100), off Princess Elizabeth Land, Stat. 29 (66°28'S, 72°41'E), 1266 m (syntypes complete, one broken in two pieces; the largest one was already dissected by Monro; 23–40 mm long, 5–9 mm wide, cephalic cage (broken) 12–21 mm long, 23–25 chaetigers).

**Figure 3.**
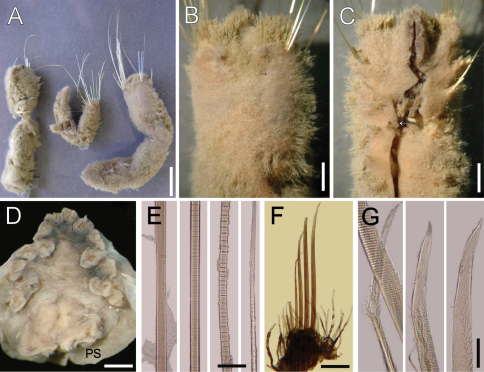
*Ilyphagus coronatus* Monro, 1939. **A** Syntypes (NHML-1941.3.3.99-100) **B** Larger syntype, anterior end, dorsal view **C** Same, anterior end, ventral view (arrow points gonopodial lobe) **D** Non-type specimen (USNM-56696), head, frontal view, palp scars and mouth directed downwards (PS: palp scar) **E** Smaller syntype, chaetiger 9, notochaetal regions **F** Same, chaetiger 5, neuropodium **G** Same, neurochaetal tips. Bars.- **A** 7 mm **B** 1.8 mm **C** 2 mm **D** 0.7 mm **E** 190 µm **F** 0.5 mm **G** 60 µm.

##### Additional material.

Three specimens (USNM-56696, LACM-AHF unnumb.), R/V Eltanin Cruise, Stat. 138 (62°00'S, 61°09'W), 1437 m, 8 Aug. 1962 (slightly damaged; larger specimen (USNM) 52 mm long, 8 mm wide, cephalic cage broken 10 mm long, 22 chaetigers). Three fragments (USNM-56697), R/V Eltanin Cruise, Stat. 480 (58°06'S, 44°56'W), 2800 m, 15 Feb. 1963 (anterior fragment, 20 mm long, 5.5 mm wide, 13 chaetigers). Two specimens (ZMH-24530), Cruise ANT/ XV-3, R/V PolarStern, South of Vestkapp, St. 48-088 (73°28.5'S, 22°30.0'W), 1681 m, 4 Feb. 1998, B. Hilbig, coll. (42–45 mm long, 10–11 mm wide, cephalic cage 7–18 mm long, 24–25 chaetigers).

##### Description.

Largest syntype cylindrical, globose, posteriorly rounded (Fig. 3A); 48 mm long, 8 mm wide, remaining cephalic cage chaetae broken, 6 mm long, 24 chaetigers. Body surface densely papillated (Fig. 3B); papillae long, filiform, tapering, or slightly capitate, with many adherent sediment particles over its basal and medial regions.

Anterior end not exposed; observed by dissection of anterior end in anterior fragment of syntypes, or other non-type specimens (USNM-56698, USNM-56696). Prostomium low cone, without eyes. No caruncle (Fig. 3D). Palps massive, as long as branchiae; palp lobes low. Lateral and dorsal lips fused; ventral lip reduced. Branchiae digitate, 14 filaments, arranged in three irregular rows: superior one with 4 filaments, two lateral groups medially placed each with 3 filaments, and two lateral basal ones with 2 filaments each; largest branchiae as long as palps.

Cephalic cage chaetae mostly broken; size relationships with body length or width unknown; syntypes with chaetae at least as long as body width and one with chaetae almost as long as body length; one very long chaetae straight, with successive constrictions but anchylosed articles perpendicular to the main shaft. Chaetiger 1 involved in cephalic cage; notochaetae of chaetiger 2 very long, thin. Chaetiger 1 with 8 notochaetae in transverse short dorsal row; neurochaetae in C-pattern, opening towards the posterior end, looking like two series, with 8 neurochaetae.

Anterior dorsal margin of first chaetiger papillated, projected anteriorly, conical, continued with the longitudinal body opening; anterior chaetigers without especially long papillae. Chaetigers 1–3 of about same length. Chaetal transition from cephalic cage to body chaetae abrupt; aristate neurospines from chaetiger 2. Gonopodial lobes in chaetiger 5, as long as neuropodial width (Fig. 3C), dark in syntypes (paler in other specimens), digitate.

Parapodial development difficult to detect due to papillae cover (USNM-56698); notopodia not detected; chaetae stem from long, rounded neuropodial lobes. Parapodia lateral; median neuropodia ventrolateral. Noto- and neuropodia close to each other.

Median notochaetae arranged in short longitudinal rows, as long as half body width, about 2 per ramus; all notochaetae multiarticulated capillaries, articles very short along most of the chaeta, distally difficult to see (Fig. 3E), hyaline. Neurochaetae anchylosed aristate spines from chaetiger 2, arranged in transverse rows, 4–5 or up to 7–8 per ramus (Fig. 5F). Both noto- and neurochaetae (USNM-56697) with distal portions rough; fibers are individually and irregularly broken off from the main axis (Fig. 3G), not hirsute.

Posterior end (USNM-56696) rounded, pygidium with anus terminal, without cirri.

##### Remarks.

*Ilyphagus coronatus* Monro, 1939 can be separated from other cigar-shaped species because of the relative size of body papillae, which appear pilose, and because its neurochaetae, despite possibly appearing hirsute due to fracture, are mostly smooth.

##### Distribution.

Only known from two localities around Antarctica, in 1200–3500 m.

#### Ilyphagus hirsutus

Monro, 1937

http://species-id.net/wiki/Ilyphagus_hirsutus

Figure 4

Ilyphagus hirsutus Monro, 1937:304–305, textfig. 22.

##### Type material.

**Central Indian Ocean, South Arabian Sea.** Holotype (NHML-1937.9.2.455), John Murray Expedition, H.E.M.S. Mabahiss, Stat**.** 133(01°25'54"S, 66°34'12"E → 01°19'42"S, 66°35'18"E), 15 Feb. 1934, 3385 m (station data after Sewell 1935).

**Figure 4.**
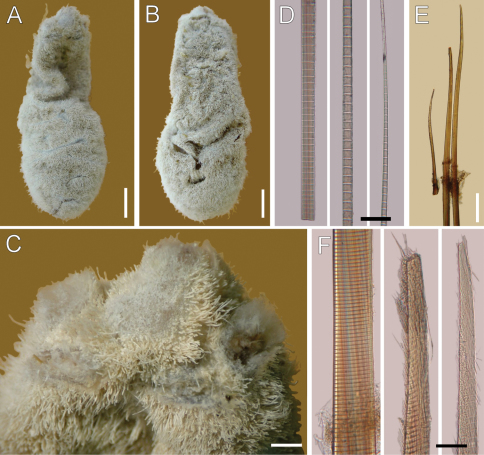
*Ilyphagus hirsutus* Monro, 1937. Holotype (NHML-1937.9.2.455). **A** Dorsal view **B** Ventral view **C** Anterior end, dorsal view **D** Chaetiger , basal, medial and distal notochaetal regions **E** Chaetiger , neurochaetae **F** Same, basal region and two hirsute tips. Bars.- **A, B** 5 mm **C** 0.2 mm **D** 45 µm **E** 0.4 mm **F** 65 µm.

##### Description.

Holotype pale, globose, widened in the posterior half (Fig. 4A, B); 37 mm long, 10 mm wide, cephalic cage chaetae broken, 19 chaetigers. Body surface densely papillated, with abundant fine sediment particles; papillae long, cylindrical, each covered by a thin layer of fine sediment particles.

Cephalic hood not exposed; specimen not dissected to avoid further damage. Cephalic cage chaetae length unknown. Chaetiger 1 involved in the cephalic cage; chaetae arranged in short rows, notochaetae dorsal, 5–7 per bundle (bases damaged, difficult to count); neurochaetae ventrolateral, 3–4 per bundle.

Anterior dorsal margin of first chaetiger projected anteriorly (Fig. 4C), large rounded lobe, bent ventrally. Anterior chaetigers without especially long papillae. Chaetiger 1 largest, chaetigers 2–3 of about the same size. Chaetal transition from cephalic cage to body chaetae abrupt; chaetiger 2 with shorter neurochaetae, directed ventrolaterally. Gonopodial lobes present in chaetiger 5, low rounded dark, displaced ventrally, and positioned towards posterior segmental margin.

Parapodia lateral; median neuropodia ventrolateral. Notopodia scarcely noticed; neuropodia thick low muscular lobes, without especially longer papillae. Noto- and neuropodia close to each other.

Median notochaetae mostly broken; one anterior notopodia with 3 notochaetae, arranged in a tuft, median notochaetae as long as 1/5 body width, about 3 per fascicle; all notochaetae multiarticulated capillaries, articles short basally, medium-sized medially, long distally (Fig. 4D). Neurochaetae probably multiarticulated capillaries in chaetiger 1; from chaetiger 2, anchylosed aristate spines (Fig. 4E), arranged in transverse rows, in two series, with about 8 chaetae per fascicle. Neurospines basally cylindrical with very short articles, medially flat, distally tapering with slightly longer articles; neurospines basally smooth, subdistally and distally with fibers separated from the main stem, giving hirsute appearance to chaetal surface (Fig. 4F).

Posterior end globose, damaged; pygidium with anus terminal, without anal cirri.

##### Remarks.

*Ilyphagus hirsutus* Monro, 1937 resembles *Ilyphagus bythincola* because of their dense coverage with fine, long papillae. They differ because *Ilyphagus hirsutus* has a projected lobe in the first neuropodia, which is not present in *Ilyphagus bythincola*, and because the neurochaetae of the former are markedly hirsute distally, whereas those in *Ilyphagus bythincola* are either distally hyaline or slightly hirsute.

##### Distribution.

Originally described from the Central Indian Ocean, in deep water (3385 m); it has not been recorded since.

#### Ilyphagus pluto

Chamberlin, 1919

http://species-id.net/wiki/Ilyphagus_pluto

Ilyphagus pluto Chamberlin, 1919:403; Hartman 1960:132.

##### Material examined.

**Off Peru.** Holotype (USNM 19721), 88 miles (141.7 km) SW Palominos Light House, R/V Albatross, Stat. 4672 (13°11'30"S, 78°18'00"W), 2845 fathoms (5206.4 m), 21 Nov. 1904.

##### Remarks.

This is a holothurian. The stout cylindrical processes forming a ‘complete closed circle'from the original description are actually tentacles surrounding the mouth. Each tentacle is short and branched, but each branch is like a wart, making them apparently crenulated. The long, typical reddish brown chaetae found penetrating the body belong to other, deep-water polychaetes, such as the aphroditid *Laetmonice*, which during dredging, frequently loose their chaetae. The holothurians belongs in the genus *Meseres*, currently in the family Synallactidae (O’Loughlin and Ahearn 2005); after O’Loughlin (2002), two species have been described from the same region: *Meseres torvus* (Théel, 1886) and *Meseres macdonaldi* Ludwig, 1894; however, the former species has an uncertain generic placement, whereas the second is retained in its genus (O’Loughlin and Ahearn 2005).

#### Ilyphagus wyvillei

(McIntosh, 1885)

http://species-id.net/wiki/Ilyphagus_wyvillei

Figure 5

Trophonia wyvillei McIntosh, 1885:366–370, pl. 44, fig. 6, pl. 23A, figs 11–14, pl. 36A, figs 5–7, pl. 37A, fig. 1.
Ilyphagus wyvillei :Hartman 1966:41–43, pl. 12, figs 7, 8 (n. comb.); Levenstein 1975:133; Detinova 1993:100–101.
Brada gravieri McIntosh, 1922:7–8, pl. 1, figs 4–6, pl. 3, fig. 1; Hartman 1966:33, pl. 9, figs 1, 2; Hartman 1978:173.

##### Type material.

**Southeastern Pacific Ocean.** Holotype (NHML-85.12.1.261), R/V Challenger Expedition, Stat. 157 (53°55'S, 108°35'E), dredged, 1950 fathoms (3568.5 m), diatom ooze, 3 Mar. 1874.

**Figure 5.**
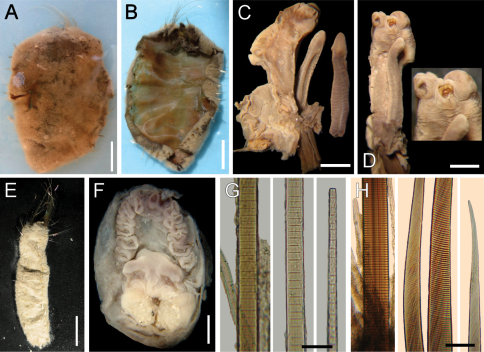
*Ilyphagus wyvillei* (McIntosh, 1885). **A** Holotype (NHML-85.12.1.261), dorsal view **B** Same, ventral view, body completely dissected and inner organs removed **C** Same, left palp and two branchial filaments **D** Same, palp and branchia, with parasite and parasite scar on palp (insert: close-up of palp tip) **E** Non-type specimen (SIORAS-unnumb.), ventral view **F** Same, head, frontal view, palps and branchiae removed **G** Holotype (NHML-85.12.1.261), chaetiger 11, basal, medial and distal notochaetal regions **H** Same, chaetiger 10, basal, medial and distal neurochaetal regions. Bars.- **A, B:** 13 mm **C** 1 mm **D** 1.7 mm **E** 10 mm **F** 2.3 mm **G** 70 µm **H** 140 µm.

##### Additional material.

**Antarctic Ocean**. Several specimens (SIORAS-unnumb.), R/V Akademik Kurchatov, Stat. 914 (56°21'S, 50°48'W), 5650–6070 m, 14 Dec. 1971 (best specimen 49 mm long, 10 mm wide, cephalic cage 24 mm long (chaetiger 2 notochaetae 18 mm long), 22 chaetigers; 11 notochaetae in chaetiger 1; two anterior fragments dissected).

##### Description.

Holotype pale brown (Fig. 5A), completely dissected mid-ventrally (Fig. 5B), internal organs and most anterior end appendages previously removed (now lost). Body sausage-shaped, anteriorly truncate, medially widened, posteriorly rounded (confirmed in non-type specimen, Fig. 5E); 63 mm long, about 30 mm wide, cephalic cage 27 mm long, 19 chaetigers. Body surface papillated; papillae abundant, cylindrical, very long, sediment particles along papillae, more abundant basally.

Anterior end dissected, most appendages now lost. Cephalic hood short, margin smooth. Prostomium flat, without eyes. No caruncle. Palps very large; one remains attached to anterior end fragment (Fig. 5C), with a distal parasite (Fig. 5D); other palp loose in container , longer than branchiae (longest remaining detached branchia 5 mm long), expanded, with a median furrow; palp lobes reduced. Branchiae cirriform, distally colorless, sessile on branchial plate, arranged in single row, in horse-shoe pattern (Fig. 5F), with 16 filaments (perhaps other four, much smaller, filaments distally, would make a secondary distal row).

Cephalic cage chaetae as long as half body length, or about as long as body width. Chaetigers 1–2 involved in the cephalic cage, chaetiger 1 with 8–9 notochaetae in a single transverse row and 11–12 neurochaetae arranged in a C-pattern, opening to posterior region; chaetiger 2 with 5–6 noto- and 9–10 neurochaetae.

Anterior dorsal margin of first chaetiger truncate, papillated; anterior chaetigers without especially long papillae. Chaetigers 1–3 becoming progressively longer. Chaetal transition from cephalic cage to body chaetae abrupt; aristate neurospines from chaetiger 3. Gonopodial lobes in chaetiger 5, short, dark digitate, mostly covered by papillae.

Parapodia poorly developed, chaetae emerge from the body wall. Parapodia lateral, median neuropodia ventrolateral. Noto- and neuropodia close to each other, without especially longer papillae, some slightly thicker papillae bordering chaetae.

Median notochaetae arranged in short transverse rows, most notochaetae broken, length relationships with body width unknown, 1–3 per ramus; all multiarticulated capillaries, articles short basally, slightly longer medially, long subdistally (tips unknown, Fig. 5G). Neurochaetae multiarticulated capillaries in chaetigers 1–2; aristate neurospines from chaetiger 3, arranged in transverse rows, 7–8 per bundle. Each neurospine with very short articles basal- and medially (Fig. 5H); distally hyaline, smooth.

Posterior end rounded; pygidium with anus ventro-terminal, without anal cirri.

##### Remarks.

*Ilyphagus wyvillei* (McIntosh, 1885) resembles *Ilyphagus bythincola* because they both have globose bodies with distally smooth aristate neurospines. They differ because *Ilyphagus wyvillei* has comparatively shorter cephalic cage chaetae than *Ilyphagus bythincola*, and because in *Ilyphagus wyvillei* there are only 16 branchiae, whereas in *Ilyphagus bythincola* there are about 40. On the other hand, *Ilyphagus wyvillei* resembles *Ilyphagus coronatus* Monro, 1939, because in their first chaetiger, neurochaetae are arranged in a C-pattern, opening to the posterior region, and by having distally smooth neurospines. However, they differ because *Ilyphagus wyvillei* has fewer chaetigers (19–22 *vs* 23–25) and a more globose body but these differences might be modified after more specimens are studied. Two other differences are probably more relevant and must be emphasized:the relative number of neurochaetae in the first chaetiger (11–12 in *Ilyphagus wyvillei*, 8 in *Ilyphagus coronatus*) , and the start of the aristate neurospines (chaetiger 2 in *Ilyphagus wyvillei*, chaetiger 3 in *Ilyphagus coronatus*).

The presence of parasitic copepods in the branchial bases of *Ilyphagus wyvillei* cannot be confirmed due to the state of the anterior end; however, one portion of a parasite is visible at one of the palps tip, and there is another deep scar in the same palp. McIntosh might have confused the attachment site, because he dissected the anterior end and branchial scars could be confused with these parasite attachment sites.

*Brada gravieri* McIntosh, 1922 might belong to the same species. There is no type material available; it is probably lost. However, the original illustrations and description noticed the lack of the cephalic cage chaetae, whereas the neurochaetae (pp 7–8) were described as translucent, smooth, devoid of transversal marks. The distal part of neurochaetae is often smooth, hyaline, but the rest of the chaetae have anchylosed articles or transverse markings throughout it. They were collected from relatively close localities but fresh material needs to be examined to clarify this .

##### Distribution. 

Originally described from the Antarctic Ocean, it has been found in abyssal depths off Western South America (Levenstein, 1975). The Bering Sea records by Levenstein (1961a:160, 1966:46), cannot be confirmed because the specimens were not found.

## Supplementary Material

XML Treatment for Ilyphagus

XML Treatment for Ilyphagus bythincola

XML Treatment for Ilyphagus coronatus

XML Treatment for Ilyphagus hirsutus

XML Treatment for Ilyphagus pluto

XML Treatment for Ilyphagus wyvillei
